# The correlation between socio-educational factors and the level of functionality in patients with Schizophrenia

**DOI:** 10.1192/j.eurpsy.2025.1561

**Published:** 2025-08-26

**Authors:** C. Babasan, M. M. Manea

**Affiliations:** 1 Psychiatry, Cluj Country Emergency Clinical Hospital, Cluj Napoca, Romania

## Abstract

**Introduction:**

The level of functioning, in patients with schizophrenia, is an essential aspect in assesing and improving their quality of life. Schizophrenia is a major contributor to severe disability in adults, as it impacts patients’ capacity to live independently, engage in social activities and pursue work or education. It is important to focus not only on reducing patients’ symptoms, but also on improving their overall functioning. There are some factors, that can improve the functional capacity of these patients, such as: family support, level of education, being employed, treatment adherence.

**Objectives:**

A 64 years old man, was diagnosed with Schizophrenia at the age of 20 years old ( 44 years ago) and he has had several hospitalizations in the Psychiatry Clinic. He is living with his 90 years old mother, has never been married and doesn’t have any children. The patient finished high school, but he doesn’t have additional studies and he has never had a job. Outside the hospitalization periods, the patient has never been compliant to the amtipsychotic treatment. The mental state exam is dominated by: complex visual and auditory hallucinations; delusional ideas of interpretation and persecution; soliloquy, stereotyped speech; bizarre, desorganized behavior; diminished self-care and self-management abilities; recent and long-term memory loss.

**Methods:**

A 65 years old man, was diagnosed with Schizophrenia at the age of 31 years old ( 34 years ago) and has had several hospitalizations in the Psychiatry Clinic. He lives with his wife, he has 3 children and 2 grandchildren. The patient finished high school and has post-secondary studies. He worked as an electrician until the age of 53 years old and then he retired due to his medical condition. The patient was compliant to the treatment for the majority of the time. The mental state exam of the patient, was dominated by: complex imperative auditory pseudohallucinations, complex visual pseudohallucinations and hallucinations, cenesthetic hallucinations; delusional ideas of persecution and interpretation, tangentiality and circumstantiality; emotional blunting, with an improvement of the symptoms over time.

**Results:**

The GAF scale was applied for both patients ( in 2024), and the difference between the two of them was significant, with the first patient scoring only 27 points, indicating a notable deterioration in his functionality. The second patient scored 58 points, indicating a much better level of functionality. The SQLS scale was also applied for both patients, the first one achieved a higher score, meaning a poor quality of life, whereas the second one obtained a lower score, meaning a better quality of life.

**Conclusions:**

The socio-educational factors play a significant importance in the quality of life, in patients with schizophrenia Mental health professionals should be aware of this factors for helping their patients to improve their functionality.

**Disclosure of Interest:**

None Declared

